# Sex-Biased Captures of Sarcosaprophagous Diptera in Carrion-Baited Traps

**DOI:** 10.1673/031.013.1401

**Published:** 2013-02-21

**Authors:** Daniel Martín-Vega, Arturo Baz

**Affiliations:** 1 Departamento de Zoología y Antropología Física, Facultad de Ciencias, Universidad de Alcalá, Ctra. Madrid-Barcelona km 33.6, 28871 Alcalá de Henares, Madrid, Spain

**Keywords:** Calliphoridae, forensic entomology, Heleomyzidae, Muscidae, Sarcophagidae, sex ratio, Piophilidae, Ulidiidae

## Abstract

The use of carrion-baited traps is a common and widely extended practice in the study of sarcosaprophagous Diptera. However, it implies different areas of bias, one of them being the different responses of males and females to carrion bait, which results in possible biased sex ratios in the captures. In the present study, the use of carrion-baited traps revealed significant female-biased captures in the families Calliphoridae, Muscidae, and Sarcophagidae, whereas the collected species of the families Piophilidae, Heleomyzidae, and Ulidiidae showed different patterns in the observed sex ratios. Possible explanations according to existing literature and the types of mating behaviors of the different families are discussed.

## Introduction

The Diptera species involved in the carriondecomposing process have been the object of several studies not only because of their important role in the good working of ecosystems, but also because of their medical and veterinary importance as vectors of a multitude of pathogens (Graczyck et al. 2001), as agents of myiasis (Hall and Wall 1995), and as forensic indicators (Amendt et al. 2011). In this sense, necrophagous species of the families Calliphoridae, Muscidae, and Sarcophagidae have been studied in detail due to their dominance in the carrion-fly communities (Hanski and Kuusela 1980; Arnaldos et al. 2001; Archer and Elgar 2003). The necrophagous species of the family Piophilidae, associated with carrion in advanced stages of decay, have received less attention, but they can also be useful in forensic studies (Martín-Vega 2011) and occasionally they can cause myiasis as well (Saleh and el Sibae 1993). Furthermore, in the carrion-fly communities, the presence of some saprophagous species, which are attracted to various kind of decomposing organic matter, cannot be ignored; hence, they are not strictly carrion-feeding species. These species do not necessarily complete their life cycles in the carrion and generally only feed upon the fluids and tissues of rotting corpses (Baz et al. 2010); however, individuals of some mainly saprophagous families like Heleomyzidae or Ulidiidae can be collected in high densities in studies of insects associated with carrion (e.g., Hwang and Turner 2005).

The use of odor and carrion bait is a widely used practice in the study of sarcosaprophagous Diptera, and there is a large variety of effective carrion-baited trap designs (e.g., Vogt et al. 1985a; Schoenly et al. 1991; Hwang and Turner 2005; Baz et al. 2007). However, the sampling of sarcosaprophagous flies with the use of carrion-baited traps could imply different areas of bias (Hwang and Turner 2005), which must be taken into account when analyzing the results of the captures. One of the possible areas of bias in this kind of study could be due to a different response of males and females of some species to carrion attractiveness, thus resulting in differences in the proportion of both sexes in the traps and an inaccurate reflection of the sex ration in the wild population. Although individuals of both sexes are attracted by decomposing odors and can be found on carrion (Archer and Elgar 2003), it is assumed that females of necrophagous flies in different stages of ovarian development are more attracted to carrion for oviposition or for the protein uptake necessary for the correct development of their ovaries (Avancini and Linhares 1988). On the contrary, the testes of male carrion flies can mature without protein feeding, and the males only visit carrion to obtain mates (Archer and Elgar 2003). As males only visit carrion to mate, different mating behaviors have been observed. Males from some species usually perch on surrounding vegetation and fly out to intercept potential mates (Archer and Elgar 2003), while males from other species meet on carcasses, defending territories or searching actively for females (Bonduriansky 2003). Perching behaviour has been observed among species from families Calliphoridae, Muscidae, and Sarcophagidae (e.g., Thomas 1950; Cook 1994; Omar et al. 1994), while males and females from families Piophilidae, Heleomyzidae ,and Ulidiidae appear to meet on oviposition sites to mate (e.g., Bonduriansky 2003; Fiedler et al. 2008; Brunel and Rull 2010b). In any case, female-biased captures of flies in carrion-baited traps are usually expected (Avancini and Linhares 1988), and such area of bias is frequently considered (e.g., Hwang and Turner 2005; Baz et al. 2007). However, it must be taken into account that every collected carrion fly species does not necessarily show a female-biased sex ratio. In any case, the causes for observed differences in sex ratios are rarely treated in detail.

The present work analyzes the observed sex ratios in a study of sarcosaprophagous flies with the use of carrion-baited traps, with the aim to determine whether or not both sexes from every species respond to carrion bait in the same way. A female-biased sex ratio would be expected in those collected species with a perching behaviour (families Calliphoridae, Muscidae, and Sarcophagidae); conversely, a non-biased sex ratio would be expected in those species that meet on carrion to mate (families Piophilidae, Heleomyzidae, and Ulidiidae). Possible causes of the different responses to carrion bait and their relation to types of mating behavior are discussed.

## Materials and Methods

Sarcosaprophagous flies were collected by means of carrion-baited traps with the aim to study the ecology of sarcosaprophagous insects in natural habitats of central Spain. The traps were made by modifying the design of the traps used by Morón and Terrón (1984) as explained by Baz et al. (2007). Traps were baited with squid, which was shown to be very effective in previous studies (Baz et al. 2007).

To study the ecology of sarcosaprophagous insects in the natural habitats of central Spain, different localities were selected based on a stratified sampling regime taking into account three variables: bioclimatical level, forest type, and soil type. This sampling regime resulted in the selection of seven main types of natural habitat distributed throughout the Community of Madrid (central Spain): 1) mesomediterranean holm oakwood (*Quercus ilex* ssp. *ballota*) on limestones; 2) id. on gypsum and marlstones; 3) id. on sands; 4) id. on granites; 5) supramediterranean holm oakwood on granites; 6) supramediterranean oakwood (*Quercus pyrenaica*) on granites and schists; 7) oromediterranean Scot's pine forests (*Pinus sylvestris*) on granites and schists. Three localities for each habitat type were selected, resulting in a total of 21 sampling sites. Detailed information about the locations and bioclimatic features of these sites can be found in Baz et al. (2010). Three traps at each site were installed and maintained from June 2006 to May 2007. Traps were baited once a month and collected for a period of seven continuous days within each month. After the seven days, the samples were carried to the laboratory and the bait was removed. A total of 63 traps remained installed for 12 months, and 756 samples were obtained. Collected specimens were conserved in alcohol or pinned and deposited in the collection of the Departement of Zoology and Physical Anthropology of the University of Alcalá.

The sex ratio, which summarizes the sex composition of a given population, was calculated for every species with more than or approximately 50 collected specimens (Table 1) as the number of males per 100 females. Furthermore, significant differences between the number of collected males and females of each species were estimated by means of Chisquare tests considering a significance level of *p* < 0.05. The sex ratios of the selected species (Table 1) were tested for differences between families and types of mating behavior (perching, considering those species whose males perch on surrounding vegetation and fly out to intercept potential mates, or meeting, considering those species whose males meet on the oviposition site and search actively for females), using analyses of variance (ANOVA). Differences were considered to be significant at the < 0.05 level.

## Results

A total number of 19,727 specimens belonging to the mainly carrion-feeding families Calliphoridae (9,295 specimens, 11 species), Muscidae (7,770 specimens, 20 species), Sarcophagidae (1536 specimens, 19 species), and Piophilidae (1,126 specimens, 8 species) were collected. The saprophagous component was mostly represented by the families Heleomyzidae (1,874 specimens, 8 species) and Ulidiidae (417 specimens, 3 species). Other saprophagous dipteran families (e.g., Dryomyzidae, Platystomatidae, or Scathophagidae) were collected in lower densities. Table 1 shows the number of males and females, sex ratios, χ^2^-tests (df = 1) for sex ratio bias, body size, and type of mating behavior of the species with more than or approximately 50 collected specimens.

**Figure 1.**
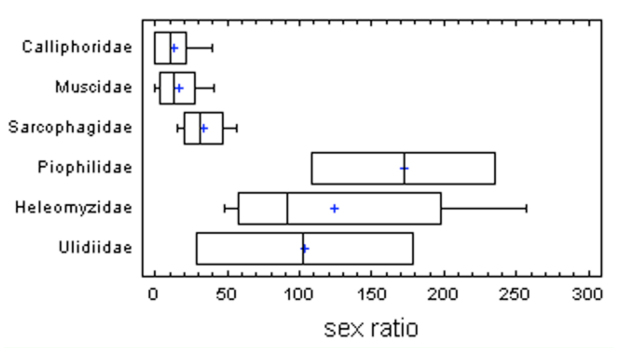
Box-and-whisker plot showing the differences in sex ratio (number of males per 100 females) between families. High quality figures are available online.

Significant female-biased captures were observed in every species in Calliphoridae, Muscidae, and Sarcophagidae (Table 1). On the contrary, the observed sex ratio was significantly different in Piophilidae, Heleomyzidae, and Ulidiidae (F = 8.10; *p* = 0.0001) (Figure 1). Within Piophilidae, the collections showed a higher number of males in the two studied species, although significant differences were only found in the species *Liopiophila varipes* (Table 1). Regarding Heleomyzidae, *Neoleria ruficeps* and *Heleomyza captiosa* showed a significant male-biased sex ratio, while *Suillia* species showed female-biased sex ratios, although differences were not significant in every species (Table 1). Finally, the three species in Ulidiidae showed three different sex ratios: *Herina gyrans* showed a significant male-biased sex ratio, *Ulidia apicalis* showed a female-biased sex ratio, and *Physiphora alceae* practically showed the same number of collected males and females (Table 1).

**Figure 2.**
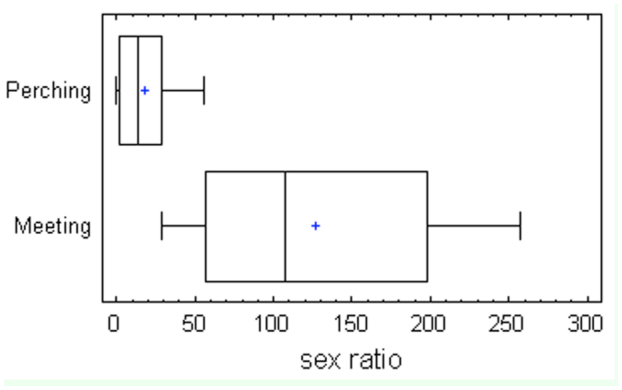
Box-and-whisker plot showing the differences in sex ratio (number of males per 100 females) between the types of mating behaviors (perching or meeting behavior). High quality figures are available online.

Differences between the two groups of families became more evident when taking into account the type of mating behavior (Figure 2). In perching species, which corresponded to species from Calliphoridae, Muscidae, and Sarcophagidae (Table 1), the observed sex ratios of the collections were clearly female-biased and significantly different (F = 38.65; *p* = 0) from those species whose males showed a meeting behavior (Figure 2), which corresponded to species from families Piophilidae, Heleomyzidae and Ulidiidae (Table 1).

## Discussion

The results showed two well-differentiated groups of sarcosaprophagous flies on the basis of the observed sex ratios. As expected, female-biased captures corresponded to those species with a perching behavior (Figure 2). However, different patterns in the observed sex ratios were found in those species with a meeting behavior (Table 1, Figure 1).

Many previous studies highlighted female-biased sex ratios of fly populations in samples from carrion-baited traps (Avancini and Linhares 1988; Hwang and Turner 2005). In the present work, female-biased samples were clearly observed in three families of sarcosaprophagous flies: Calliphoridae, Muscidae, and Sarcophagidae (Table 1, Figure 1). These three families are dominant in the carrion fly communities (Hanski and Kuusela 1980; Arnaldos et al. 2001; Archer and Elgar 2003), although, due to their importance in medicine and veterinary science, the majority of works testing different carrion baits were done on Calliphoridae (e.g., Morris et al. 1998; Hayes et al. 1999). In these studies, a greater attractiveness of carrion baits for female calliphorid flies was observed independent of the age of bait (Vogt and Woodburn 1994) or the climatic conditions (Vogt et al. 1985b). Furthermore, gravid females and females in the early stages of ovarian development were more attracted to carrion than females from other age categories (Hayes et al. 1999). Gravid females use the carrion as a substrate for oviposition, while females in the early stages of ovarian development could obtain from carrion a protein uptake for the correct development of ovaries (Avancini and Linhares 1988; Hayes et al. 1999).

Regarding the other dominant families in the carrion fly assemblages, female-biased captures were reported in Muscidae (Vogt et al. 1985a; Patitucci et al. 2010), but no significant differences in the age composition of the collected females were observed (Tyndale-Biscoe and Hughes 1969). In the case of Sarcophagidae, several studies showed a higher proportion of females captured by using different types of carrion-baited traps (Martínez-Sánchez et al. 2000; Romera et al. 2003). However, Aspoas (1994) captured more males than females using traps baited with fish carcasses, although he later obtained a sex ratio of 1:1 from the laboratory cultures. Dodge and Seago (1954) obtained a female-biased sex ratio in their carrion-baited trap captures of Sarcophagidae, although for some species the captures were male-biased, including some species of genus *Ravinia* Robineu-Desvoidy. One species, *R. pernix* (Harris), from this genus was collected in the present study, but in a density lower than 50 individuals; therefore, this species was not included in the analysis (see Material and Methods). Remarkably, *R. pernix* was the only Sarcophagidae species that showed a significant male-biased sex ratio in the present study (data not shown).

It is assumed that females of necrophagous flies are more attracted to carrion due to a greater development of their olfactory receptors, which have been proposed to be located in insects at the antenna sensilla (Greenberg 1970; Shanbhag et al. 1999). Sukontason et al. (2004) did not find differences in the morphology and number of each type of sensillum between sexes of some species of Calliphoridae, Muscidae and Sarcophagidae, but found a greater number of sensory pits in the antenna sensilla of females than in males of some Sarcophagidae and Calliphoridae species. The sensory pits seem to work as olfactory receptors (Wasserman and Itagaki 2003) and are very numerous in Sarcophagidae females (Slifer and Sekhon 1964), but they seem to be particularly scarce in both sexes in Muscidae (Lewis 1971; Bay and Pitts 1976; Been et al. 1988). Perhaps the reason for the female-biased captures of necrophagous flies with carrion baits lies not only in a different degree of development in the olfactory receptors of both sexes, but also in the mating behavior of the different species. Observations in the field have not revealed acts of mating of Calliphoridae and Muscidae (Omar et al. 1994) or Sacophagidae (Thomas 1950) on carcasses. Males from these families appear to show a perching behavior, so they are usually not observed on carcasses or odor sources, but downwind from them, where they intercept and attempt to mate with flies passing by (Cook 1994). In accordance with this behavior, it has been observed that males can be collected by net more frequently around carrion (Dodge and Seago 1954; Martínez-Sánchez et al. 2000). Moreover, Dodge and Seago (1954) collected by net several male and female couples *in copula* around carrion-baited traps, and collected by trap high numbers of single females from Calliphoridae, Muscidae, and Sarcophagidae. This explanation according to the perching mating behaviour fits with the sex ratio observed in the present study for these three families (Figure 2). However, there was a complete absence of *Pollenia* males in the collections of the present study (Table 1). Although *Pollenia* species are primarily considered as parasites or predators of earthworms, they are frequently attracted to carrion. Their function in the decomposition processes remains unclear (Baz et al. 2007). Using the same type of carrion-baited traps as the present study, Baz et al. (2007) collected both males and females of *Pollenia* species, but the sex ratio for the most abundant species was female-biased.

On the contrary, the observed sex ratios seem to be unbiased in those species with a meeting mating behavior (Figure 2). This unbiased sex ratio is found in Piophilidae (Figure 1), which is commonly present on carrion but in lower numbers than other necrophagous flies (Velásquez et al. 2010). In accordance with the results of the present study (Table 1), the sex ratio in Piophilidae has been observed to be unbiased or male-biased (Bonduriansky and Brooks 1999). Mating, courtship, and oviposition of piophilid flies occur on the carcass, where males of some species usually defend territories, charging any fly within the surrounding area (Bonduriansky 2003). Males are sometimes more abundant than females on carcasses, and they often harass them (Bonduriansky and Brooks 1999; Bonduriansky 2003; Fiedler et al. 2008). One species that shows harassing behavior and a male-biased sex ratio on carcasses is *L. varipes* (Bonduriansky and Brooks 1999); a male-biased sex ratio for this species was also observed in the present study (Table 1). Other piophilid species, however, do not show harassing behavior, and their sex ratio can be unbiased on carrion (Bonduriansky and Brooks 1999), such as *P. nigrimana* in the present study (Table 1). Another species of genus *Prochyliza* Walker, *P. xanthostoma* Walker, has shown unbiased sex ratios on carcasses (Bonduriansky and Brooks 1999). Males from this species show a meeting mating behavior, but they do not appear to harass females (Bonduriansky and Brooks 1999; Bonduriansky 2003).

A meeting mating behaviour was observed in some species from Heleomyzidae (Tuno et al. 2003; Fiedler et al. 2008). Males from the necrophagous genus *Neoleria* Malloch defend territories and usually harass females, resulting in a male-biased sex ratio on carcasses (Fiedler et al. 2008). The significant male-biased sex ratio observed in the *N. ruficeps* in the present study (Table 1) was thus in accordance with such observations. On the other hand, a meeting mating behavior of males, as well as a male-biased sex ratio on ovipositional substrates, was also observed in *Suillia* species (Tuno et al. 2003). In the present study, *Suillia* species showed an unbiased or significantly female-biased sex ratio (Table 1). It is worth recalling, however, that the preferred ovipositional substrate of *Suillia* females is not carrion, but basidiocarps, where males wait to mate with mature females, excluding conspecific invaders (Tuno et al. 2003). Adults of different *Suillia* species, including some of the species collected in the present study (Table 1), were collected on carcasses (e.g., Castillo Miralbes 2002) or with carrion-baited traps (e.g., Hanski and Nuorteva 1975), but there are no data about the use of carrion as a breeding site by the species of this genus. However, it is difficult to assign a specific trophic regime for one species. For example, necrophagous species such as some Calliphoridae and Piophilidae could eventually breed on decomposing vegetal organic matter (Norris 1965; Zuska and Laštovka 1965). On the contrary, coprophagous species of Scathophagidae could be frequently collected in association with carrion, and some species could breed on decomposing animal tissues (see Hanski and Kuusela 1980). A wide range of breeding substrates are particularly shown in the present species of Muscidae (see Gregor et al. 2002 for a review). Hence, carrion could serve as an additional food resource for *Suillia* species and eventually as an oviposition site, as *Suillia* occur with other saprophagous insects that feed on different decomposing tissues including carrion, although they do not usually complete their life cycle on carcasses (Baz et al. 2010). Both males and females could be thus attracted to carrion, but the higher proportion of *Suillia* females could be more related to the use of carrion as an additional protein source, as is the case in females in early stages of ovarian development from other carrion fly families (Avancini and Linhares 1988).

It is difficult to explain, however, the different sex ratios observed in the three collected species of Ulidiidae (Table 1). Ulidiid flies were collected on carcasses (e.g., Castillo Miralbes 2002) and with carrion-baited traps (e.g. Hwang and Turner 2005), but most species of Ulidiidae have saprophagous larvae (Allen and Foote 1967). There is little published information on the mating behavior of Ulidiidae, but observations on *Euxesta bilimeki* and *Pseudodyscrasis scutellaris*, whose larvae breed on rotting leaves of agave plants, showed that both males and females could be found on the decomposing organic matter, which serves as oviposition site (Brunel and Rull 2010a, 2010b). Although males were not found defending territories, they continuously patrolled the oviposition site searching for females, harassing them and performing complex courtship movements (Brunel and Rull 2010b). There are no data about the development of larvae of the collected species of Ulidiidae (Table 1) in carrion, and the biology and natural history of most ulidiid species remain unknown. More studies should be done in order to determine if the observed male-biased or an unbiased sex ratio in some of the present species (Table 1) indicates that those species could mate on carrion with certain frequency.

Fly species could show different responses to carrion-baited traps depending on the way they use carrion. Sarcosaprophagous species make the most of an ephemeral but valuable resource like carrion. Calliphorid, muscid, and sarcophagid females could exploit carrion as a nutritive resource for the development and maturation of their reproductive system, as well as a breeding site for their offspring, which usually dominates the carrion insect community in terms of number and drives the decomposition process. Nevertheless, carrion is a limited habitat, an ecological island supporting a whole community of insects and other organisms (Beaver 1977; Braack 1987). In light of this scenario, other fly species could also exploit benefits from such a valuable resource, expanding their use to a site for mating, giving even more sense to the definition of carrion as a habitat. This meeting mating behavior on decomposing organic matter usually involves complex patterns of courtship, harassment, or recognition between sexes (e.g., Bonduriansky 2003), which contrast enormously with the perching behavior of males from the dominant families, which intercept and try to mount any passing fly resembling a potential mate in the vicinity of a carcass, whether it is another male or an individual from another species (Omar et al. 1994). Such variability in the mating behavior is a reflection of the species diversity associated with carrion and their different strategies to exploit such a resource, which permits coexistence (see Braack 1987). Knowing and inventorying the diversity of sarcosaprophagous fly species associated with carrion is necessary from a biological and applied point of view, and carrion-baited traps are very valuable tools in this kind of study. However, it is essential to bear in mind the possible areas of bias when carrion-baited traps are used (see Hwang and Turner 2005). It is evident that conclusions regarding the sex composition of the wild population should not be made. In fact, laboratory observations show that the sex ratio of emerged individuals is approximately 1:1 (El-Dessouki and Stein 1980; Arnaldos et al. 2001). On the other hand, monitoring the abundance and diversity of sarcosaprophagous Diptera with the use of carrion-baited traps can be useful to investigate their population dynamics and to evaluate different strategies for control of myiasis in livestock (Vogt and Woodburn 1994), as well as to provide further information on the necrophagous fauna associated with carrion, which can aid in forensic investigations (see Amendt et al. 2011 for a review). Therefore, the knowledge of the sex-related differences in the responses to carrion-bait by the different taxa could serve as an additional proof to evaluate the efficiency of the chosen sampling baits and methods in studies about sarcosaprophagous Diptera.

**Table 1.**
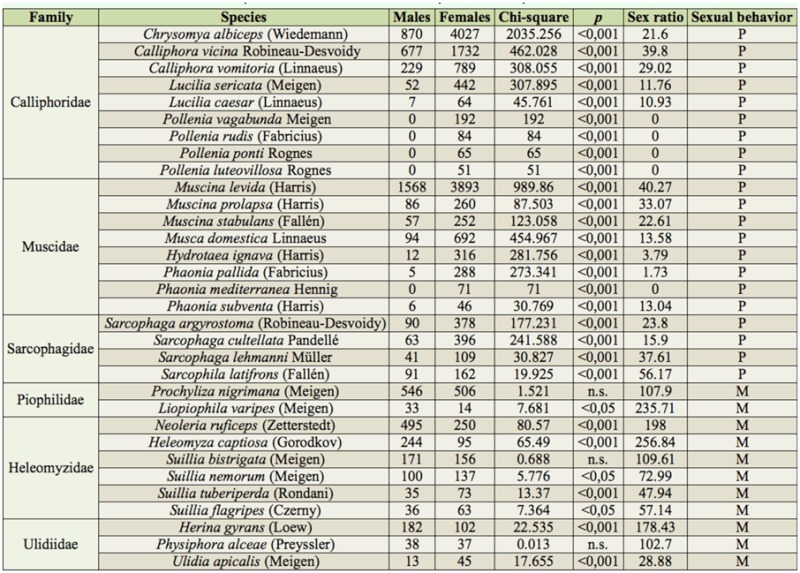
Sex-ratios of the families and species most abundant of the present study.
